# The role of *TNF *genetic variants and the interaction with cigarette smoking for gastric cancer risk: a nested case-control study

**DOI:** 10.1186/1471-2407-9-238

**Published:** 2009-07-17

**Authors:** Jae Jeong Yang, Kwang-Pil Ko, Lisa Y Cho, Aesun Shin, Jin Gwack, Soung-Hoon Chang, Hai-Rim Shin, Keun-Young Yoo, Daehee Kang, Sue K Park

**Affiliations:** 1 Department of Preventive Medicine, Seoul National University College of Medicine, Seoul, Korea; 2 Division of Epidemiology and Health Index, Center for Genome Science, Korea Center for Disease Control and Prevention, Seoul, Korea; 3 National Cancer Control Research Institute, National Cancer Center, Goyang, Korea; 4 Department of Preventive Medicine, Konkuk University, Chungju, Korea; 5 Seoul National University Cancer Research Institute, Seoul, Korea; 6 Department of Molecular Medicine and Biopharmaceutical Sciences, Graduate School of Convergence Science and Technology and College of Medicine, Seoul National University, Seoul, Korea

## Abstract

**Background:**

The aim of this study was to investigate the role of *TNF *genetic variants and the combined effect between *TNF *gene and cigarette smoking in the development of gastric cancer in the Korean population.

**Methods:**

We selected 84 incident gastric cancer cases and 336 matched controls nested within the Korean Multi-Center Cancer Cohort. Six SNPs on the *TNF *gene, *TNF*-α-238 G/A, -308 G/A, -857 C/T, -863 C/A, -1031 T/C, and *TNF*-β 252 A/G were genotyped. The ORs (95% CIs) were calculated using unconditional logistic regression model to detect each SNP and haplotype-pair effects for gastric cancer. The combined effects between the *TNF *gene and smoking on gastric cancer risk were also evaluated. Multi dimensionality reduction (MDR) analyses were performed to explore the potential *TNF *gene-gene interactions.

**Results:**

*TNF*-α-857 C/T containing the T allele was significantly associated with an increased risk of gastric cancer and a linear trend effect was observed in the additive model (OR = 1.6, 95% CI 1.0–2.5 for CT genotype; OR = 2.6, 95% CI 1.0–6.4 for TT genotype). All haplotype-pairs that contained TCT or CCC of *TNF*-α-1031 T/C, *TNF*-α-863 C/A, and *TNF*-α-857 C/T were associated with a significantly higher risk for gastric cancer only among smokers. In the MDR analysis, regardless of smoking status, *TNF*-α-857 C/T was included in the first list of SNPs with a significant main effect.

**Conclusion:**

*TNF*-α-857 C/T polymorphism may play an independent role in gastric carcinogenesis and the risk for gastric cancer by *TNF *genetic effect is pronounced by cigarette smoking.

## Background

Gastric cancer is a multi-factorial disease that requires the study of environmental, genetics, and host-related factors in order to understand its pathology. The strongest risk factor for gastric cancer is Helicobacter pylori (*H. pylori*) which is labeled a group I human gastric cancer carcinogen by the International Agency for Research on Cancer [1]. However, a high prevalence of *H. pylori *infection does not always result in a high incidence of gastric cancer. Only a small portion of *H. pylori *infected persons have gastric cancer, thereby suggesting that other susceptible factors such as genetic variants or environmental differences must additionally be involved in gastric carcinogenesis.

Genetic variants of inflammation-related cytokines are a potential risk factor because *H. pylori *infection induces chronic inflammation in gastric mucosa which is a critical step in gastric carcinogenesis. One of the major cytokines associated with *H. pylori *infection is the tumor necrosis factor (*TNF*) expressed by *TNF*-α and *TNF*-β genes [2-6]. Many studies have extensively investigated the association between *TNF *and gastric cancer [5-15]. Although studies have reported *TNF *can modify the risk of gastric cancer [5,8-11], the exact role of *TNF *as a gastric carcinogen is still controversial.

In terms of environmental factors, cigarette smoking has been suggested to play a crucial role in increasing the risk of gastric cancer. Previous epidemiologic studies indicated that cigarette smoking was an independent risk factor for gastric cancer development [16-21]. Moreover, cigarette smoking may promote gastric cancer development by activating systemic inflammation [22]. Hamajima et al. proposed that cigarette smoking and cytokines such as *TNF*-α and *Interleukin*-1B may change normal mucosa to *H. pylori *infected mucosa that may be a first step towards gastric carcinogenesis [22].

Genetic variants of *TNF *cytokines and cigarette smoking may play a role in gastric carcinogenesis. They seem to play independent or synergetic roles in gastric cancer but the mechanism is still unclear. Thus, we hypothesized that genetic variants of *TNF *underlies the association with gastric cancer risk and/or their combined effect with cigarette smoking may modify the risk of gastric cancer. The aim of this study was to assess the role of *TNF *gene variants and the combined effect between *TNF *genes and cigarette smoking in the development of gastric cancer in the Korean population.

## Methods

### Study population and data collection

In this population-based nested case-control study, we selected subjects from the Korean Multi-Center Cancer Cohort (KMCC), a prospective cohort of participants (N = 19,688) recruited from four urban and rural areas in Korea (Haman, Chungju, Uljin, and Youngil) [23]. The participants signed a consent form and completed a detailed standardized interview-based questionnaire on general lifestyle, diet, medical history, and other environmental factors, and blood and spot urine samples were collected and stored at -70°C and -20°C, respectively. All information was from baseline survey questionnaires that were collected before gastric cancer diagnosis. Cancer ascertainment was identified by passive surveillance through record linkages to the national cancer registry, the national death certificate, and the health insurance medical records databases [23]. A total of 136 gastric cancer cases were ascertained in December 2002. Of the 136 cases, 36 cases diagnosed before recruitment and 16 cases that lacked DNA for genotyping were excluded. Eligible non-cancer controls were randomly selected from the KMCC population. Four controls were matched to each gastric cancer case by four variables that were age (± 5 years), sex, residential district, and enrollment year. Finally, 84 gastric cancer cases and 336 controls were included in this study.

### Genotyping

*TNF*-α gene was genotyped for five single nucleotide polymorphisms (SNPs) that were -238 G/A(rs361525), -308 G/A(rs1800629), -857 C/T(rs1799724), -863 C/A(rs1800630), and -1031 T/C(rs1799964). These five polymorphisms were located in the promoter region of *TNF*-α gene. The *TNF*-β gene that is synonymous with Lymphotoxin-α (LTA) was genotyped for 252 A/G (rs909253) SNP. Genotyping was performed using SNaPshot method. Ten samples were randomly selected and genotyped for each SNP to check for reliability. All assays were 100% concordant. More than 95% of the total subjects were successfully genotyped for all SNPs.

### *H. pylori *serum assays

Serum was evaluated to identify IgG antibodies specific for *H. pylori *and seropositivity of CagA/VacA by Helico Blot 2.1™ (MP Biomedicals Asia Pacific, Singapore) according to the manufacturer's protocol. [24,25]. The sensitivity and specificity for detecting seropositivity of *H. pylori*, CagA and VacA of Helico Blot 2.1™ were very high (for sensitivity, 99%, 99% and 93%, respectively; for specificity, 98%, 90% and 88%, respectively) [26].

### Statistical analysis

Unconditional logistic regression model was used to calculate the odds ratios (ORs) and 95% confidence intervals (95% CIs) of selected characteristics such as follow time (years), *H. pylori *seropositivity, alcohol consumption, and cigarette smoking status for gastric cancer risk. To evaluate significant covariates, we reviewed the literature and conducted logistic regression analysis; smoking status, *H. pylori *infection, and CagA seropositivity were selected. Because of the significant association between age/sex and smoking status, we also included age and sex as major covariates. In the analysis, all five covariates were selected adjusted variables, and smoking status was also selected as a stratified variable.

The Hardy-Weinberg equilibrium assessed allele frequencies using the chi-square test. Both single SNPs and haplotype analysis were performed. To detect each SNP effect for gastric cancer, three genetic models, dominant, co-dominant and additive model, were used. Tests for significance were computed after adjusting for age, sex, smoking status, *H. pylori *infection, and CagA seropositivity. Also, in order to correct for multiple comparisons, false discovery rate (FDR) that controls the expected proportion of incorrectly rejected null hypotheses (type I errors) was computed [27].

To evaluate putative haplotype blocks, linkage disequilibrium (LD) between loci expressing genetic variation was analyzed using Haploview 4.0. Moreover, pairwise Lewontin's D' and r^2 ^values were calculated with 95% CIs when the genotype frequencies were in Hardy-Weinberg equilibrium [28]. Three haplotype blocks were generated which included the following: 1) only three *TNF*-α SNPs -857, -863, and -1031; 2) three *TNF*-α SNPs in block 1 and *TNF*-β 252; and 3) all six SNPs on *TNF*-α and *TNF*-β were generated by the Haploview software using the method suggested by Gabriel et al [29] (Additional file 1).

Based on haplotype analysis, each individual haplotype-pair was estimated with haplotype frequencies greater than 5%. We stratified according to smoking status to assess the combined effect of the *TNF *gene and smoking for gastric cancer. To evaluate the association between risk for gastric cancer and *TNF *haplotype-pairs effects, unconditional logistic regression model was used to calculate the ORs and 95% CIs with adjustment for age, sex, *H. pylori *infection and CagA seropositivity.

Multi dimensionality reduction (MDR) analyses were performed to explore the potential *TNF *gene-gene interactions. MDR is a non-parametric data mining approach for detecting a potential gene-gene or gene-environment interaction and providing selective models of high-order combination of genes [30,31]. MDR methodology is described in detail elsewhere [30-32]. *TNF *gene-gene interaction was evaluated using a naïve Bayes classifier in the context of a 10-fold cross-validation to estimate the test accuracy (TA) of the best 1-, 2-, 3-, and 4- factor models (Additional file 2). The best single interaction model was selected by maximized TA, lowest prediction error or higher cross-validation consistency (CVC). Significance was evaluated using a sign test and was determined at the 0.05 level. Finally, we conducted logistic regression to assess the association between genotype combinations suggested by MDR and gastric cancer with the MDR ORs and 95% CIs. According to stratification by smoking status, MDR ORs were also calculated after adjusting for age, sex, *H. pylori *infection and CagA seropositivity. All statistical analyses using logistic regression models were performed with SAS version 9.1 and MDR analyses were implemented using MDR software version 11.0 (available at http://www.epistasis.org).

### IRB approval

The study protocols for the KMCC study and the present nested case-control study were approved by the institutional review boards of Seoul National University Hospital and the National Cancer Center of Korea (H-0110-084-002, and C-0603-161-170, respectively).

## Results

Table 1 presents the odds ratios and 95% CIs of the selected characteristics for gastric cancer risk. There was no significant difference between cases and controls according to age, gender, median follow up period (FU years), education level, *H. pylori *infection, CagA/VacA seropositivity, and alcohol consumption. All smoking related factors were associated with an increased risk for gastric cancer. Current smokers had a significantly higher risk for gastric cancer compared to never smokers (OR = 1.8, 95% CI 1.0–3.2). Moreover, a dose-response relationship between smoking intensity and gastric cancer risk was found. Smoking more cigarettes per day in addition to longer smoking duration showed a significantly higher risk compared to never smokers (OR = 3.9, 95% CI 1.4–10.7 for more than 20 cigarettes per day; OR = 1.9, 95% CI 1.1–3.5 for more than 20 years). 41 or more pack-years of smoking was related to a statistically increased risk for gastric cancer (OR = 2.3, 95% CI 1.1–4.9).

**Table 1:** Odds ratios and 95% CIs for gastric cancer risk according to basic characteristics of study subjects in a nested case-control study within the KMCC 19,688 enrolled cohort members.

	Case (N = 84)	Control (N = 336)	OR (95% CI)
**Sex**			
Male	59 (70)	236 (70)	1.0 (reference)
Female	25 (30)	100 (30)	1.0 (0.6–1.7)
**Age^a^**			
≤ 63 years old	43 (51)	176 (52)	1.0 (reference)
> 63 years old	41 (49)	160 (48)	1.1 (0.7–1.7)
**Follow up^b^**			
≤ 6 years for FU	47 (56)	188 (56)	1.0 (reference)
> 6 years for FU	37 (44)	148 (44)	1.0 (0.6–1.6)
**Education level**			
without any schooling	22 (26)	98 (29)	1.0 (reference)
schooling	62 (74)	237 (71)	1.2 (0.7–2.0)
***H. pylori *infection**			
negative (-)	12 (14)	48 (14)	1.0 (reference)
positive (+)	72 (86)	288 (86)	1.0 (0.5–2.0)
**CagA**			
negative (-)	11 (13)	54 (16)	1.0 (reference)
positive (+)	73 (87)	282 (84)	1.3 (0.6–2.5)
**VacA**			
negative (-)	36 (43)	156 (46)	1.0 (reference)
positive (+)	48 (57)	180 (54)	1.2 (0.7–1.9)
**Drink status^c^**			
Never drinkers	38 (45)	144 (43)	1.0 (reference)
Ever drinkers	46 (55)	188 (57)	0.9 (0.6–1.5)
**Smoking status**			
Never smokers	26 (31)	141 (42)	1.0 (reference)
Ex smokers	21 (25)	82 (25)	1.4 (0.8–2.6)
Current smokers	37 (44)	111 (33)	1.8 (1.0–3.2)*
**Cigarette per day**			
Never smokers	26 (31)	141 (42)	1.0 (reference)
20 ≤	15 (18)	59 (18)	1.4 (0.7–2.8)
>20	8 (9)	11 (3)	3.9 (1.4–10.7)**
unreported	35 (42)	125 (37)	1.5 (0.9–2.7)
**Smoking duration (years)**			
Never smokers	26 (31)	141 (42)	1.0 (reference)
20 ≤	5 (6)	29 (9)	0.9 (0.3–2.6)
>20	32 (38)	88 (26)	1.9 (1.1–3.5)*
unreported	21 (25)	78 (23)	1.5 (0.8–2.8)
**Pack-Year^d^**			
Never smokers	26 (31)	141 (42)	1.0 (reference)
20<	5 (6)	14 (4)	1.9 (0.6–5.8)
20–40	8 (10)	40 (12)	1.1 (0.5–2.6)
≥ 41	14 (17)	33 (10)	2.3 (1.1–4.9)*
unreported	31 (36)	108 (32)	1.6 (0.9–2.8)

* p < 0.05, ** p < 0.01

a. Median age for cases and controls was 63 years. Age ranged from 40.0 to 83.0 years old.

b. The median year for follow up was 6 years. Number of follow-up years ranged from 2 to 8 years.

c. Ever drinkers defined as former and current drinkers.

d. Pack years was calculated by the equation, (number of cigarettes smoked per day × number of years smoked)/20

Table 2 shows the distribution of *TNF *polymorphisms among cases and controls and the ORs (95% CIs) for gastric cancer risk in relation to *TNF *genetic polymorphisms. All genotype frequencies of each SNP did not deviate from the Hardy-Weinberg equilibrium (*p *> 0.1). In single SNPs analysis, *TNF*-α-857 C/T containing the T allele was significantly associated with an increased risk of gastric cancer. CT genotype of *TNF*-α-857 showed a 1.7 fold increased risk for gastric cancer in the co-dominant and dominant models that were statistically significant (95% CI 1.0–2.8, identically in both models). However the TT genotype of *TNF*-α-857 showed an insignificantly increased risk in the co-dominant model (OR = 1.7, 95% CI 0.3–9.2). In the additive model which showed a linear trend effect, the CT genotype of *TNF*-α-857 showed a 1.6 fold increased risk for gastric cancer (95% CI 1.0–2.5) and the TT genotype of *TNF*-α-857 had a 2.6 fold increased risk (95% CI 1.0–6.4). Both increase risks for the T allele in the additive model were statistically significant. Under multiple comparison tests by FDR, all *p*-values of *TNF *SNPs in each additive model were greater than 0.2.

**Table 2:** The association between genetic polymorphisms of *TNF *and risk of gastric cancer in a nested case-control study within the KMCC 19,688 enrolled cohort members.

				OR (95% CI)^a^			
					
Gene	Genotype	Case	Control	Co-dominant	Dominant	Additive	raw *p *value^b^
***TNF *β 252 A/G (rs909253)**	**AA**	23 (29)	83 (26)	1.0 (reference)	1.0 (reference)	1.0 (reference)	
	**AG**	43 (54)	170 (54)	0.9 (0.5–1.6)	0.9 (0.5–1.5)	0.9 (0.6–1.3)	0.5110
	**GG**	13 (17)	61 (20)	0.8 (0.4–1.7)		0.8 (0.4–1.7)	
***TNF *α-1031 T/C (rs1799964)**	**TT**	55 (66)	205 (63)	1.0 (reference)	1.0 (reference)	1.0 (reference)	
	**TC**	25 (30)	109 (34)	0.8 (0.5–1.4)	0.8 (0.5–1.4)	0.9 (0.6–1.4)	0.5915
	**CC**	3 (4)	10 (3)	1.1 (0.3–4.3)		0.9 (0.3–1.9)	
***TNF *α-863 C/A (rs1800630)**	**CC**	64 (77)	227 (70)	1.0 (reference)	1.0 (reference)	1.0 (reference)	
	**CA**	16 (19)	90 (28)	0.6 (0.3–1.1)	0.7 (0.4–1.2)	0.8 (0.5–1.3)	0.3416
	**AA**	3 (4)	6 (2)	1.6 (0.4–6.9)		0.6 (0.2–1.7)	
***TNF *α-857 C/T (rs1799724)**	**CC**	49 (58)	227 (70)	1.0 (reference)	1.0 (reference)	1.0 (reference)	
	**CT**	33 (39)	92 (28)	1.7 (1.0–2.8)*	1.7 (1.0–2.8)*	1.6 (1.0–2.5)*	**0.0466**
	**TT**	2 (3)	6 (2)	1.7 (0.3–9.2)		2.6 (1.0–6.4)*	
***TNF *α-308 G/A (rs1800629)^c^**	**GG**	75 (90)	288 (89)	1.0 (reference)	1.0 (reference)	1.0 (reference)	
	**GA**	8 (10)	34 (11)	0.8 (0.4–1.9)	0.8 (0.3–1.7)	0.7 (0.3–1.6)	0.4439
	**AA**	-	-	-		0.5 (0.1–2.5)	
***TNF *a-238 G/A (rs361525)^c^**	**GG**	73 (88)	305 (92)	1.0 (reference)	1.0 (reference)	1.0 (reference)	
	**GA**	10 (12)	26 (8)	1.6 (0.7–3.6)	1.6 (0.7–3.6)	1.6 (0.7–3.6)	0.2333
	**AA**	-	-	-		2.5 (0.5–12.1)	

* p < 0.05, ** p < 0.01

a. Adjusted for age, sex, smoking status (Never vs. Ever), *H. Pylori *infection, and CagA seropositivity.

b. Calculated by likelihood ratio test in each additive model. FDR adjusted p-values for all SNPs were not significant (p > 0.2).

c. Excluded mutant genotype (AA genotype) frequency less than 1% or totally 0%.

Table 3 shows the association between haplotype-pairs of *TNF *gene and gastric cancer risk according to smoking status. In block 1 that is composed of *TNF*-α-1031 T/C, *TNF*-α-863 C/A and *TNF*-α-857 C/T (D' = 1.0 and r^2 ^= 0.037), compared to TCC-TCC without a variant allele, TCC-TCT showed a significantly increased risk for gastric cancer among total subject and smokers (OR = 2.1, 95% CI 1.1–3.9; OR = 2.6, 95% CI 1.2–5.8; respectively) but was not associated with gastric cancer risk among non-smokers (OR = 1.5, 95% CI 0.5–4.0). TCC-CCC was associated with an increased risk only among smokers (OR = 2.9, 95% CI 1.0–8.9). Similar results were represented in block 2 that is composed of the three *TNF*-α SNPs in block 1 and *TNF*-β 252 A/G (D' = 1.0 and r^2 ^= 0.184), and block 3 that is composed of all six *TNF *SNPs (D' = 1.0 and r^2 ^= 0.068). Compared to the most frequent haplotype-pairs GTCC-GTCC in block 2, ATCT-XXXX or ACCC-XXXX that contained TCT or CCC of *TNF*-α-1031 T/C, *TNF*-α-863 C/A and *TNF*-α-857 C/T showed a significantly higher risk among smokers but not among non-smokers (OR = 2.5, 95% CI 1.0–6.8 for ATCT haplotype-pairs; OR = 4.1, 95% CI 1.2–14.2 for ACCC haplotype-pairs). Particularly, among non-smokers, other haplotype-pairs which did not contain ATCT or ACCC were associated with a significantly reduced risk for gastric cancer compared to the reference haplotype-pairs (OR = 0.2, 95% CI 0.1–0.7). Similarly for block 1 and 2, ATCTGG or ACCCXX haplotype-pairs that contained TCT or CCC of *TNF*-α-1031 T/C, *TNF*-α-863 C/A and *TNF*-α-857 C/T were also associated with a significantly higher risk for gastric cancer only among smokers (OR = 3.1, 95% CI 1.0–10.0 for ATCTGG; OR = 4.7, 95% CI 1.2–18.2 for ACCCXX).

**Table 3:** Haplotype-pairs effects of *TNF *genetic polymorphisms for gastric cancer stratified by smoking status in a nested case-control study within the KMCC 19,688 enrolled cohort members.

Haplotype pairs	Total			Non-smokers			Smokers		
	
	Case	Control	OR (95% CI)^a^	Case	Control	OR (95% CI)^b^	Case	Control	OR (95% CI)^b^
**Block1: *TNF *α-1031, -863, -857^c^**									
TCC-TCC	25 (34)	132 (44)	1.0 (reference)	11 (50)	58 (45)	1.0 (reference)	14 (27)	73 (44)	1.0 (reference)
TCC-**TCT**	28 (38)	72 (24)	2.1 (1.1–3.9)*	9 (41)	33 (26)	1.5 (0.5–4.0)	19 (37)	38 (23)	2.6 (1.2–5.8)*
TCC-**CCC**	7 (10)	20 (7)	1.7 (0.6–4.6)	0 (0)	8 (6)	0.3 (0.0–5.6)^d^	7 (14)	12 (7)	2.9 (1.0–8.9)*
TCC-CAC	13 (18)	73 (25)	0.9 (0.4–1.9)	2 (9)	29 (23)	0.3 (0.1–1.7)	11 (22)	44 (26)	1.3 (0.5–3.1)

**Block2: *TNF *β 252, α-1031, -863, -857**									
GTCC-GTCC	15 (18)	71 (21)	1.0 (reference)	9 (35)	30 (22)	1.0 (reference)	6 (10)	40 (21)	1.0 (reference)
A**TCT**-XXXX ^e^	35 (42)	101 (31)	1.7 (0.8–3.3)	13 (50)	41 (30)	1.0 (0.4–2.9)	22 (38)	59 (31)	2.5 (1.0–6.8)*
A**CCC**-XXXX ^f^	8 (9)	24 (7)	1.5 (0.6–4.2)	0 (0)	11 (8)	0.1 (0.0–2.6)^d^	8 (14)	13 (7)	4.1 (1.2–14.2)**
Others	26 (31)	137 (41)	0.9 (0.4–1.8)	4 (15)	57 (41)	0.2 (0.1–0.7)*	22 (38)	80 (42)	1.9 (0.7–5.0)

**Block3: all *TNF *6 SNPs^g^**									
GTCCGG* GTCCGG	10 (12)	57 (17)	1.0 (reference)	6 (23)	24 (17)	1.0 (reference)	4 (7)	32 (17)	1.0 (reference)
A**TCT**GG-XXXXXX ^h^	34 (40)	91 (27)	2.1 (0.9–4.7)	13 (50)	37 (26)	1.4 (0.5–4.3)	21 (36)	53 (27)	3.1 (1.0–10.0)*
A**CCC**XX-XXXXXX ^i^	8 (10)	24 (7)	1.8 (0.6–5.2)	0 (0)	11 (8)	0.2 (0.0–3.2)^d^	8 (14)	13 (7)	4.7 (1.2–18.2)*
ACACGG-XXXXXX ^j^	12 (14)	55 (16)	1.1 (0.5–2.9)	2 (8)	21 (15)	0.3 (0.1–2.0)	10 (17)	34 (17)	2.3 (0.7–8.2)
Others	20 (24)	108 (33)	1.0 (0.4–2.4)	5 (19)	47 (34)	0.4 (0.1–1.6)	15 (26)	61 (32)	1.9 (0.6–6.3)

* p < 0.05, ** p < 0.01

a. Adjusted for age, sex, *H. Pylori *infection, CagA seropositivity, and smoking status (Never vs. Ever).

b. Adjusted for age, sex, *H. Pylori *infection, and CagA seropositivity.

c. If frequency of haplotype pairs among controls was less than 5%, we did not include in the logit model.

d. Logit estimate for OR and 95% CI.

e. Haplotype pairs were ATCT-GTCC, ATCC-ATCT, ATCT-ATCT and ATCT-ACAC.

f. Haplotype pairs were ATCC-ACCC, ACCC-ACAC and ACCC-GTCC.

g. All SNPs, *TNF *β 252, *TNF *α-1031, *TNF *α-863, *TNF *α-857, *TNF *α-238 and *TNF *α-308, were included to estimate individual haplotypes.

h. Haplotype-pairs were ATCTGG-GTCCGG, ATCCGG-ATCTGG, ATCTGG-ATCTGG, ATCTGG-ACCCAG, ATCTGG-ACACGG and ATCTGG-ATCCAA.

i. Haplotype-pairs were ACCCGG-ACACGG, ACCCAG-ACACGG, ACCCAC-GTCCGG, ACCCAC-GTCCGA and ATCCGG-ACCCAG.

j. Haplotype-pairs were ACACGG-ACACGG, ACACGG-GTCCGG and ACACGG-GTCCGA.

Table 4 presents the summary of MDR analysis and association between high risk genotype combinations suggested by MDR analysis and gastric cancer risk. We selected separate best models according to smoking status. The best MDR model for *TNF *SNPs among total subjects included *TNF*-α-1031 T/C, *TNF*-α-863 C/A, *TNF*-α-857 C/T and *TNF*-α-308. This model had maximum TA of 0.602 and maximum CVC of 9/10 (*p *= 0.0015). The global OR adjusted for the age, sex, smoking status, *H. pylori *infection, and CagA seropositivity was 2.2 (95% CI 1.3–3.6). Regardless of smoking status, *TNF*-α-857 C/T was included in the first list of SNPs with a significant main effect. *TNF*-α-857 model among total subjects had TA of 0.554 and CVC of 9/10 but was not significant at the level of 0.0799. Similar results were shown among non-smokers and smokers. Among non-smokers, the model included only *TNF*-α-857 and had a maximum CVC of 9/10 and a higher prediction for gastric cancer risk of (OR = 1.7, 95% CI 1.0–2.7) though showing minimum TA of 0.603 (*p *= 0.0636). In contrast, although the model included *TNF*-β 252 A/G, *TNF*-α-1031 T/C, *TNF*-α-857 C/T, and *TNF*-α-308 G/A had a higher CVC of 8/10 and maximum TA of 0.697 (*p *= 0.0009), it had a lower predictability for gastric cancer risk since the adjusted global OR by this model was not significant (OR = 1.2, 95% CI 0.7–2.0). The best model of smokers was selected with *TNF*-α-1031 T/C, *TNF*-α-863 C/A and *TNF*-α-857 C/T. This model had a maximum CVC of 10/10, TA of 0.615 (*p *= 0.0023) and adjusted global OR of 2.0 (95% CI 1.2–3.3). Although the model included *TNF*-α-1031 T/C, *TNF*-α-863 C/A, *TNF*-α-857 C/T, and *TNF*-α-308 G/A had maximum TA with the lowest *p *value, its CVC was relatively low (7/10).

**Table 4:** MDR analysis for *TNF *SNPs and risk of gastric cancer associated with selected combination in MDR stratified by smoking status in a nested case-control study within the KMCC 19,688 enrolled cohort members.

		Test accuracy	CVC^a^	*p value *^b^	Case	Control	OR (95% CI)^c^
**Total**	**α-857**	0.554	9/10	0.0799	49 (58)	227 (70)	1.0 (reference)
					35 (42)	98 (30)	1.7 (1.0–2.8)*
	**α-857, α-308**	0.577	6/10	0.0225	42 (51)	207 (65)	1.0 (reference)
					41 (49)	110 (35)	1.8 (1.1–3.0)*
	**α-863, α-857, α-308**	0.591	6/10	0.0044	42 (51)	215 (70)	1.0 (reference)
					40 (49)	94 (30)	2.2 (1.3–3.7)**
	**α-1031, α-863, α-857, α-308^d^**	0.602	9/10	0.0015	36 (44)	193 (63)	1.0 (reference)
					46 (56)	113 (37)	2.2 (1.3–3.6)**

**Non-smokers**	**α-857**	0.603	9/10	0.0636	49 (58)	227 (70)	1.0 (reference)
					35 (42)	98 (30)	1.7 (1.0–2.7)*
	**α-857, α-308**	0.664	6/10	0.0038	42 (51)	207 (65)	1.0 (reference)
					41 (49)	110 (35)	1.8 (1.1–3.0)*
	**β 252, α-857, α-308**	0.680	6/10	0.0030	34 (43)	158 (52)	1.0 (reference)
					45 (57)	143 (48)	1.5 (0.9–2.4)
	**β 252, α-1031, α-857, α-308**	0.697	8/10	0.0009	40 (51)	165 (56)	1.0 (reference)
					39 (49)	130 (44)	1.2 (0.7–2.0)

**Smokers**	**α-857**	0.554	6/10	0.1863	51 (61)	233 (72)	1.0 (reference)
					33 (39)	92 (28)	1.6 (1.0–2.7)*
	**α-1031, α-857**	0.570	7/10	0.0130	32 (39)	151 (48)	1.0 (reference)
					51 (61)	165 (52)	1.4 (0.9–2.4)
	**α-1031, α-863, α-857^d^**	0.615	10/10	0.0023	42 (51)	210 (68)	1.0 (reference)
					40 (49)	101 (32)	2.0 (1.2–3.3)**
	**α-1031, α-863, α-857, α-308**	0.662	7/10	0.0015	43 (52)	215 (70)	1.0 (reference)
					39 (48)	91 (30)	2.1 (1.3-3.5)**

* p < 0.05, ** p < 0.01

a. Cross-validation consistency

b. *P*-values were estimated by sign test.

c. Among total subjects, the ORs (95% CIs) were adjusted for age, sex, smoking status (Never vs. Ever), *H. Pylori *infection, and CagA seropositivity, and after stratification for smoking status, the ORs (95% CIs) were adjusted for age, sex, *H. Pylori *infection, and CagA seropositivity, except smoking status.

d. Overall best model attributed by the MDR method

## Discussion

In our study, *TNF*-α-857 C/T genetic variant containing the T allele was associated with a significantly increased risk for gastric cancer. SNP-SNP interaction in the *TNF *gene including *TNF*-α-857 C/T or *TNF*-α-1031 T/C genetic variant was associated with a risk for gastric cancer among smokers but not among non-smokers. Moreover this effect of the *TNF*-α-857 C/T genetic variant was ascertained in both haplotype and MDR analysis.

Previous studies reported that *TNF *gene polymorphisms modified the risk of gastric cancer [5,8-11], while others did not find a significant association [6,7,12-15]. This inconsistency may be due to the small number of cases, differences in ethnic populations and SNP selection. Two recent meta-analyses assessed the association between *TNF*-α polymorphisms and risk of gastric cancer [33,34]. According to these papers, *TNF*-α-308 polymorphism had a significantly increased risk for gastric cancer but *TNF*-α-857 polymorphism only showed a marginally significant risk due to the small number of studies. In contrast to the meta-analysis, our results indicated that *TNF*-α-857 polymorphism was associated with a significantly increased risk for gastric cancer but other SNPs including *TNF*-α-308 did not show a significant association. Our findings about the single SNP effect of *TNF*-α-857 polymorphism can help clarify results of the previous meta-analyses. Inconsistency in *TNF*-α-308 polymorphism may be due to an ethnic difference since *TNF*-α-308 AA polymorphism is rare in the East Asian population. Also, we were not able to clearly evaluate *TNF*-α-308 AA polymorphism and gastric cancer because none of our gastric cancer cases had the *TNF*-α-308 AA polymorphism.

In haplotype-pairs and MDR analysis, we observed a genetic combined effect among *TNF *SNPs. Individual haplotype-pairs including *TNF*-α-1031, -863 and -857 were consistently associated with a significantly increased risk of gastric cancer. Compared to the single SNP effect of the *TNF*-α-857 polymorphism, a greater odds ratio for haplotype-pairs including *TNF*-α-857 indicated that a synergistic interaction among *TNF *SNPs was more strongly associated with gastric cancer development. These results were nearly replicated in the MDR analysis. Our consistent findings from the different statistical methodologies are quite meaningful.

*TNF *cytokine may interact with cigarette smoking to promote gastric cancer development. Biologically, cigarette smoking can activate systemic inflammations [35] and augment the level of *TNF*-α through changes in inflammatory markers or cytokine level in animal models [36-38]. Although these findings have not been fully reproduced in humans, several papers suggest an indirect connection between cigarette smoking and *TNF *gene in the etiology of gastric cancer [39-42]. Some studies reported that circulating concentrations of *TNF*-α were increased and higher *TNF*-α level was associated with such diseases in smokers compared to non-smokers [39-42]. In one of these studies, higher *TNF*-α levels were presented among smokers, especially subjects with the 857 T allele and rare haplotype of the *TNF*-α promoter [39]. Similar to our findings, the study concluded that *TNF*-α-857 polymorphism was especially susceptible to the hazards of smoking and the *TNF *cytokine was strongly affected by cigarette smoking.

The combined effect between smoking and *TNF *gene was definitely expressed in our haplotype-pairs and MDR analysis. Haplotype-pairs formed with *TNF*-α-1031, -863 and -857 were associated with a significantly greater increased risk for gastric cancer only among smokers but not among non-smokers. Both single SNP and haplotype-pair effect of *TNF*-α-857 T allele were related with an increased risk among smokers. Haplotype-pairs including *TNF*-α-1031 and -863 genetic variation that did not show significance for single SNP effect were also associated with a significantly increased risk of gastric cancer among smokers only. In contrast to *TNF*-α-857, *TNF*-α-1031 and -863 may interact mutually based on haplotype specificity and not allele specificity, and this effect may be strongly affected by smoking. If only allele specificity by high-producing or mutant alleles plays a role as risk factors for gastric cancer, haplotype-pairs composed of a greater number of high-producing or mutant alleles may have an even greater risk. Based on this hypothesis, CAT haplotype should be the most powerful risk factor for gastric cancer development and CAC haplotype composed of two high-producing alleles should show a greater risk than CCC or TCT haplotype that includes only one high-producing allele. However, we were not able to fully test this hypothesis because only four haplotypes, TCC, TCT, CCC and CAC, were observed in our population. Considering our results, independent allele specificity of *TNF*-α-857 T and mutual haplotype specificity of *TNF*-α-1031 and -863 may be more important risk factors rather than the total number of high-producing alleles, especially for smokers. Moreover, these results were reproduced in all haploblocks regardless of different SNP combinations and were nearly replicated in the MDR analysis. This supports our conclusion that the interaction between the *TNF *gene and smoking may play a crucial role in the etiology of gastric cancer.

Although, to our knowledge, this is the first study to report an interaction between the *TNF* gene and cigarette smoking on gastric cancer development, our study had several limitations. First, because of a small number of gastric cancer cases and small sample size, we did not have sufficient statistical power and were not able to stratify on different factors. Second, although we had information on cardiac or non-cardiac cancer of gastric cancer cases, because of our small number of cases, we were not able to observe a difference for gastric cancer risk according to cancer type. Moreover, we did not collect information on cancer histology. Finally, a part of SNPs which is related to signal pathway (NF-κB) from *H. pylori *to cytokine gene expression were genotyped. Other cytokines, such as *interleukin*s and GM-CSF, which are involved in NF-κB pathway, were not considered for analysis so we examined only a small portion of signal pathway for gastric carcinogenesis.

In spite of these limitations, our study had several strengths. This is a population-based, nested case-control study that is free of biases that are common in retrospective studies. Additionally, we matched cases and controls according to basic confounders, such as age and sex, and significant confounding factors selected in the full model. Furthermore, we used various approaches to detect the potential association between genetic and environmental factors on gastric cancer, and derived consistent results through different approaches. Finally, the minor allele frequencies (MAFs) of all cytokine genes analyzed in our study showed very similar results in the Korean, Chinese, and Japanese Hapmap projects [43,44] and thus, our results are applicable to most East-Asian populations. On the basis of this study results, we will be able to make more conclusive evidence in future studies.

## Conclusion

This study demonstrates that *TNF*-α-857 C/T polymorphism may play an independent role in gastric carcinogenesis and the gene-gene interaction of *TNF *also affects gastric cancer development. The combined effect between *TNF *gene and cigarette smoking can be a major risk factor for gastric cancer. Tailored smoking cessation programs should be targeted for smokers with *TNF *genetic variants. Additional studies with a greater number of cases and information about gastric cancer type and various genes will allow us to conduct stratified analysis to obtain more detailed results that will further clarify the role of genetic and environment factors on gastric cancer.

## Competing interests

The authors declare that they have no competing interests.

## Authors' contributions

All the authors participated in the design, conduct, and analysis of the study, and approved the final version of this paper.

## Pre-publication history

The pre-publication history for this paper can be accessed here:

http://www.biomedcentral.com/1471-2407/9/238/prepub

## Supplementary Material

Additional file 1 **LD blocks of *TNF *gene: A) only three *TNF*-α SNPs -857, -863, and -1031; B) three *TNF*-α SNPs in block 1 and *TNF*-β 252; and C) all six SNPs on *TNF*-α and *TNF*-β**. These LD blocks are generated by the Haploview software using the method suggested by Gabriel et al. Click here for file

Additional file 2 **Dendrograms for *TNF *genetic variants among A) total subjects, B) smokers, and C) non-smokers modeled by MDR analysis**. These dendrograms show high-order combination of *TNF *genes and indicate *TNF *gene-gene interaction. Click here for file
