# Supplementary figures and images for: The role of TNF genetic variants and the interaction with cigarette smoking for gastric cancer risk: a nested case-control study
Source: BMC Cancer. 2009 Jul 17;9:238. doi: 10.1186/1471-2407-9-238 (PMC2725140; doi:10.1186/1471-2407-9-238)

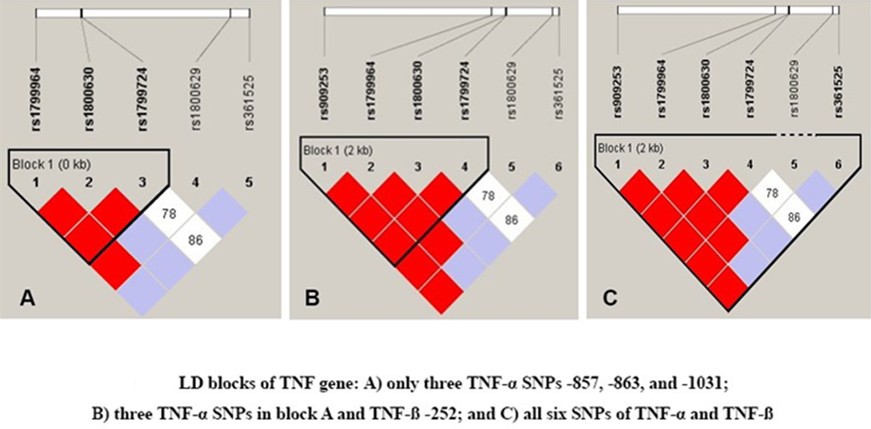

Supplement: Additional file 1 — LD blocks of TNF gene: A) only three TNF-α SNPs -857, -863, and -1031; B) three TNF-α SNPs in block 1 and TNF-β 252; and C) all six SNPs on TNF-α and TNF-β. These LD blocks are generated by the Haploview software using the method suggested by Gabriel et al. [file 1471-2407-9-238-S1.jpeg]

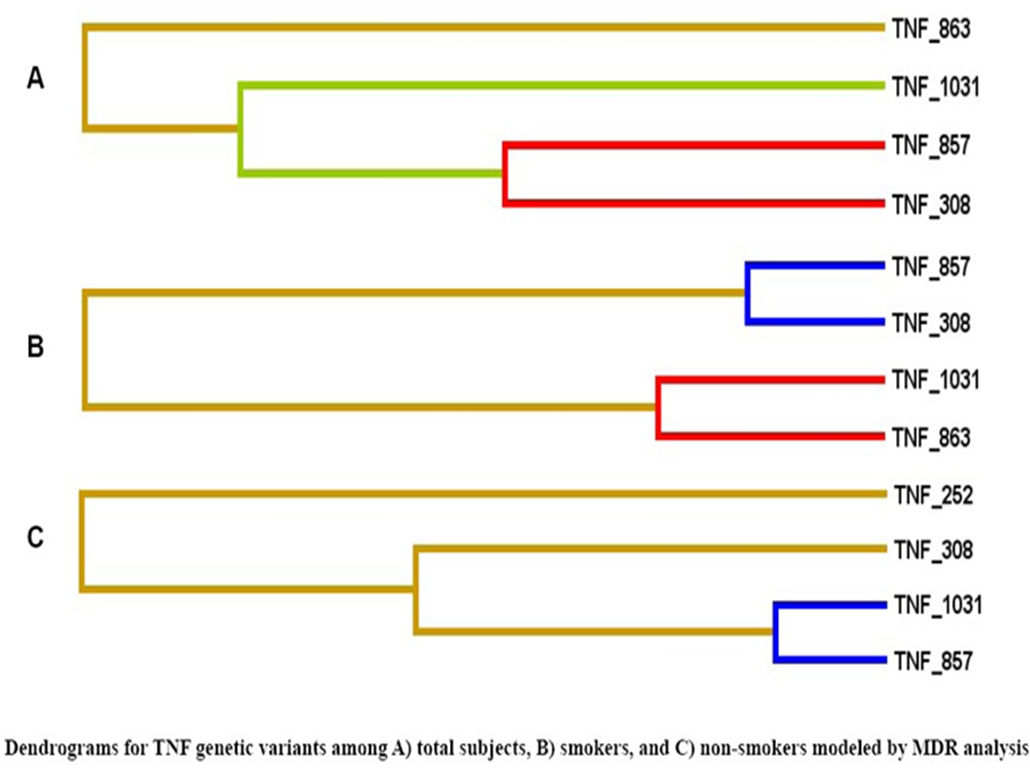

Supplement: Additional file 2 — Dendrograms for TNF genetic variants among A) total subjects, B) smokers, and C) non-smokers modeled by MDR analysis. These dendrograms show high-order combination of TNF genes and indicate TNF gene-gene interaction. [file 1471-2407-9-238-S2.png]
